# Effects of grazing on grassland or wood-pasture, slaughtering age and ageing time on meat production and quality in Podolian young bulls

**DOI:** 10.1080/01652176.2024.2381544

**Published:** 2024-08-01

**Authors:** Francesco Giannico, Despoina Karatosidi, Claudia Carbonara, Marco Ragni, Simona Tarricone, Anna Caputi Jambrenghi, Luigi Tedone, Maria Antonietta Colonna

**Affiliations:** a Department of Veterinary Medicine, University of Bari Aldo Moro, Valenzano, Bari, Italy; b Research Institute of Animal Science, Hellenic Agricultural Organization-DIMITRA, Giannitsa, Pella, Greece; c Department of Soil, Plant and Food Sciences, University of Bari Aldo Moro, Bari, Italy

**Keywords:** Fatty acid profile, grassland bulls, Podolian cattle, meat quality, wood-pasture

## Abstract

Podolian cattle is an autochthonous breed well adapted to the harsh semi-arid environments of the Southern Italy regions; the extensive rearing system used for these indigenous animals is based on grazing on spontaneous pastures, such as grasslands or wood pastures These grazing systems respect animal welfare and enrich animal products with characteristics closely related to the feeding system and the farming environment. The aim of the present study was to characterize the nutritional value of a forage crop and a wood-pasture and to evaluate the effects of grazing by Podolian young bulls on the performances and meat quality in relation to the age at slaughter (14 or 18 months) and to the ageing time of meat (3, 9 or 14 days). The metabolizable energy and the gas production were greater in April and June for both pasture systems. Young bulls raised on the grassland showed greater slaughter weights (*p* < 0.05) as compared to those fed on the woodland system, at both the slaughtering ages. The Warner Bratzler Shear (WBS) force values for raw and cooked meat were not influenced by the pasture system but they significantly (*p* < 0.01) decreased in relation to the ageing time in all the groups. Ageing markedly (*p* < 0.05) increased the malondialdehyde (MDA) concentration from 3 to 14 days of storage, regardless of the pasture system and the slaughtering age. The n-6/n-3 polyunsaturated fatty acid ratio of meat was markedly lower in grassland animals, regardless of the age of slaughter. In conclusion, 18 months old grassland beef showed better performances and yield of meat cuts. Ageing for 9 days positively affected meat WBS without increasing MDA concentration.

## Introduction

In recent years, free-range smallholder beef farming is often practiced extensively with animal feeding relying only on natural pastures and crop residues with little concentrate supplementation (Nyambali et al. 2023). The effectiveness of this beef production system depends on the quality and availability of natural pasture grasses which vary throughout the seasons. In regions with a temperate climate, natural pastures represent a sustainable feeding strategy for livestock (Bhandari et al. 2016; Karatosidi et al. 2023). Pasture-feeding lowers animal management costs and manure production, with great benefits for the preservation of the silvo-pastoral environment. Furthermore, meat obtained from grassland ruminants is increasingly appreciated by the modern consumer due to its ‘green-healthy image’ (Boogaard et al. 2011), which plays an important role in purchasing choices (Van Wezemael et al. 2014; Musto et al. 2016).

In Mediterranean ecosystems, with regard to shrubs, the nutritional values of leaves of different species depends on their physical and chemical characteristics. The young leaves are more tender and nourishing than the mature ones, that are harder and drier (Topalidou et al. 2021), for which ruminants, and especially cattle, show little preference. Herbaceous plants begin their growth in early spring and dry up much earlier in summer than bushy plants, while during summer semi-deciduous shrubs lose some of their leaves; dimorphic species have summer leaves that show a different morphology and chemical composition than winter leaves (Palacio et al. 2007). Finally, evergreen shrubs and trees show significant differences in several leaf characteristics. The nutritional value of plant tissues depends on several factors, such as the nitrogen and the water contained in the plant tissue, the species and tissue hardness (Topalidou et al. 2021).

Extensive use of grazing may be difficult in Southern Italy areas characterized by low summer rainfall and poor pasture quality (Marino et al. 2006a, Cosentino et al. 2023); moreover, natural pastures show a high variability in the floristic composition. Climatic stressors, warming and drought, influence several plant physiological processes, thus affecting the nutritional value of pastures (Martins-Noguerol et al. 2023). It has been reported that drought exerts a greater impact on grasses compared to forbs (Wellstein et al. 2017). The balance in plant communities might affect pasture quality, since the vegetal species differ in their palatability and nutritive value (Lee 2018), thus limiting their feeding potential in livestock production. Moreover, according to the National Forest Inventory (2015), forests cover an area of 189,086 hectares in the Apulian region (Southern Italy), which represents 9.7% and 39% of the regional and national territory, respectively. Apulia is, therefore, one of the least wooded regions in Italy, and it requires a careful forest management to support livestock production (De Laurentis et al. 2021).

Besides the economic, ecological, managerial, and productive advantages, animal grazing contributes to the maintenance of biodiversity, leads to the increase in the number of pabular and non-pabular species, reduces the accumulation of necro mass and soil losses and allows better control of invasive species. Furthermore, grazing can be practiced in areas where it is impossible to intervene mechanically and, finally, provides benefits to animal welfare.

Podolian cattle are well adapted to the harsh semi-arid environments with poor vegetation; skin pigmentation, well developed dewlap and sturdy hooves, disease resistance and longevity are some examples of the anatomical and physiological adaptations of this autochthonous breed to the environment (Napolitano et al. 2002; Bragaglio et al. 2018).

These indigenous animals are able to display their own ethogram in extensive rearing systems which are able to minimize the use of chemicals and provide them with a sustainable production environment (Napolitano et al. 2001). These conditions positively affect animal welfare and influence the fat content, fatty acid composition, and sensorial properties of meat from ruminants (Ponnampalam et al. 2024).

An extensive system may affect meat quality, as previously stated by authors who found that grazing induced darker meat colour in young bulls (Muir et al. 1998), which influences meat appearance and consumers’ purchasing decisions. Consumers also prefer the lower fat content and the better fatty acid profile of grassland beef (Van Wezemael et al. 2014). Previous studies have highlighted that meat from grassland animals shows better nutritional features and healthier fat as compared to feedlot finished animals (French et al. 2000; Davis et al. 2022; Nogoy et al. 2022; Ponnampalam et al. 2024).

The aim of the present study was to characterize the nutritional value of a forage crop and a wood-­pasture and to evaluate the effects of grazing by Podolian young bulls on the performances and meat quality in relation to the age at slaughter (14 or 18 months) and to the ageing time of meat (3, 9 or 14 days).

## Materials and methods

### Animal rearing

According to the Directive 2010/63/EU, all animals were ethically and responsibly managed.

The study was carried out during February–October in a dairy farm located in Mottola (in the province of Taranto, Apulia Region, Italy; Latitude: 40.688564, Longitude: 16.967435, 300 m a.s.l.). Forty Podolian calves born from parents registered in the relevant herdbooks of the breed were used. According to the traditional farming system for this autochthonous breed, the calves were exclusively milk-fed, suckling from the cows until they reached the age of about 10 months in February. Then they were separated, and the calves were allowed to have free access to pasture.

The calves were weighed (341.34 ± 1.50 kg) and randomly assigned to one of the two organic extensive grazing systems: a group grazed on a forage crop production system (FC) while the other grazed on a wood pasture (WP). Within each grazing system, two sub-groups of young bulls (*N* = 10) were made, which were slaughtered at the age of 14 or 18 months, respectively. The four groups of calves were kept outdoors throughout the year, as there were no dangerous environmental conditions in the selected farm.

The forage crop production system was characterized by a mixture of oat (70%, *Avena sativa*), vetch (20%, *Vicia sativa*) and clover (10%, *Trifolium alexandrinum*). The crop was sown during October of the year before the experiment: half of the crop was grazed, while the other half was harvested to produce hay, that was fed to animals from July to October.

The wood-pasture system was characterized by the presence of *Quercus trojana* and other deciduous species commonly present in deciduous forests habitats (*Teucrio siculi-Quercetum trojanae*) (Biondi et al. 2004).

During the day, the animals were allowed to graze, while at housing, in the evening, they were supplemented with an organic concentrated feed (1% of live weight/day/bull) containing oat (32%), barley (32%), field bean (32%), dehydrated carobs (3%) and mineral premix (1%). The chemical composition and fatty acid profile of the concentrate is shown in Table 1.

**Table 1.** Chemical composition and fatty acid profile of the concentrate feed.

% on dry matter	%
Moisture	9.8
Protein	16.38
Fat	2.79
Ash	6.10
Crude fibre	9.54
NDF	26.04
ADF	10.14
ADL	1.43
AIA	0.82
**% of fatty acid methyl esters**	
C14:0	0.29
C15:0	0.12
C16:0	15.08
C17:0	0.32
C18:0	1.90
C15:1	0.16
C16:1	0.12
C17:1	0.37
C18:1 n-7	1.27
C18:2 n-6 t	0.50
C18:2 n-6 c	0.43
C18:3 n-3	1.94
C20:2 n-6	0.24
C20:3 n-6	0.33
EPA	0.65
C21:5 n-3	0.28
C22:5 n-3	0.06
C22:5 n-6	0.90
C22:6 n-3	0.26
C24:1 n-9	0.25

NDF neutral detergent fibre; ADF acid detergent fibre; ADL acid detergent lignin; AIA acid insoluble ash.

### Pasture sampling and analysis

The floristic composition of pastures was defined following the method described by Stohlgren et al. (1995). Three modified—Whittaker vegetation plots (Stohlgren et al. 1995) were established.

Inside each plot of 20 × 50 m (1000 m^2^) 1 subplot of 5 × 20 m (100 m^2^) was chosen. For each of these, 2 subplots of 2 × 5 m (10 m^2^) and 10 subplots of 0.5 × 2 m (1 m^2^) were identified. In each 1 m^2^ subplots, all plant species were identified and for each of them the cover percentage was visually estimated. For each of these species, only the mean cover value is reported in the species list of the entire plot. The floristic analysis was based on the floras by Pignatti (2017). The nomenclature was standardized using the checklist of the Italian Flora by Conti et al. (2005). The biomass sampling was carried out on February 19^th^, April 23^rd^, June 15^th^, August 28^th^ and October 22^nd^ of the same year.

The list of botanical species and their relative abundance (lower percentage value – upper percentage value) of the woodland pasture is shown in Table 2. The distribution of the main families of the botanical species during the sampling periods is reported in Table 3.

**Table 2.** List of species of the wood pasture and their presence during the months of sampling.

APIACEAE	February	April	June	August	October
*Eryngium campestre* L.	X	X	X	–	X
*Ferula communis* L.	X	X	X	–	X
*Thapsia garganica* L.	X	X	X	–	X
LILIACEAE				–	
*Allium tenuiflorum* Ten.	X	X	X	–	X
*Asparagus acutifolius* L.	X	X	X	X	X
*Asphodeline lutea* (L.) Rchb.	X	X	X	X	X
*Asphodelus ramosus* L. subsp. Ramosus	X	X	–	–	X
RANUNCULACEAE					
*Nigella damascena* L.	–	X	X	–	–
REDEDACEAE					
*Reseda lutea* L.	–	X	X	–	–
RUBIACEAE					
*Galium corrudifolium* Vill.	X	X	X	–	X
SCROPHULARIACEAE					
*Bartsia trixago* L.	X	X	X	–	–
*Verbascum macrurum* Ten.	X	X	X	X	X
ASTERACEE					
*Carduus pycnocephalus* L. subsp. Pycnocephalus	X	X	X	–	–
*Carthamus lanatus* L. subsp. Lanatus	X	X	X	X	X
*Centaurea deusta* Ten.	X	X	X	X	X
*Chondrilla juncea* L.	X	X	X	X	X
*Crepis corymbosa* Ten.	X	X	X	X	X
*Lactuca serriola* L.	–	X	X	X	X
*Picris hieracioides* L.	X	X	X	X	X
*Scolymus hispanicus* L.	X	X	X	–	X
*Scolymus maculatus* L.	X	X	X	–	X
*Scorzonera villosa* Scop. subsp. columnae	X	X	X	X	X
*Silybum marianum* (L.) Gaertn.	X	X	X	X	X
*Sonchus asper* (L.) Hill	X	X	X	–	X
BORAGINACEAE					
*Echium asperrimum* Lam.	X	X	X	X	X
*Borago officinalis* L.	–	X	X	–	X
BRASSICACEAE					
*Erysimum crassistylum* Presl	X	X	X	–	X
CAMPANULACEE					
*Campanula scheuchzeri* Vill.	–	X	–	–	–
CONVOLVULACEAE					
*Convolvulus arvensis* L.	X	X	X	X	X
EUPHORBIACEAE					
*Euphorbia exigua* L	X	X	X	X	X
FABACEAE					
*Astragalus hamosus* L.	X	X	X	–	X
*Lathyrus cicera* L.	X	X	X	–	X
*Medicago minima* (L.) L.	X	X	X	–	X
*Trifolium campestre* Schreber	X	X	X	–	X
*Trifolium scabrum* L. subsp. scabrum	X	X	–	–	X
LABIATEAE					
*Thymus spinulosus* Ten.	X	X	X	X	X
MALVACEAE					
*Malva sylvestris* L.	X	X	X	–	–
MIRINACEAE					
*Ardisia crenata* Sims.	X	X	X	X	X
PAPAVERACEAE					
*Papaver rhoeas* L.	X	X	X	–	–
PLANTAGINACEAE					
*Plantago bellardi* All.	X	X	X	X	X
POACEAE					
*Bromus erectus* Huds. subsp. erectus	X	X	X	X	X
*Dactylis glomerata* L. subsp. hispanica (Roth) Nyman	X	X	X	X	X
*Dasypyrum villosum* (L.) Borbás	X	X	X	X	X
*Triticum ovatum* (L.) Raspail	X	X	X	X	X

**Table 3.** Cover index of the wood pasture during the sampling periods.

	February	April	June	August	October
Not palatable	51	82	42.5	21	30
Compositae	46	64	36	20	34
Legumes	4	10	7	0	13
Poaceae	10	17	13	7	19
Others	9	17	11	2	5

All the samples were oven-dried at 65 °C for 48 h and weighed to determine dry matter (DM); subsequently, they were ground in a hammer mill and analysed in triplicate using the procedures suggested by Association of Official Analytical Chemists (AOAC) (2004). The total lipids were extracted using a 2:1 chloroform/methanol (v/v) solution to determine the fatty acid profile (Folch et al. 1957). The fatty acids were then methylated using a KOH/methanol 2 N solution (Christie 1982) and analysed by gas chromatography (Shimadzu GC-17A) using a silicone-glass capillary column (70% Cyanopropyl Polysilphenylene-siloxane BPX 70 by Thermo Scientific, length = 60 m, internal diameter = 0.25 mm, film thickness = 0.25 µm). The starting temperature was 135 °C for 7 min, then increased by 4 °C/min up to 210 °C. Fatty acids were identified by comparison of retention times to authentic standards for percentage area normalization. Fatty acids were expressed as a percentage (wt/wt) of total methylated fatty acids.

The pasture samples were analysed for the *in vitro* gas production according to the procedures described by Menke and Steingass (1988). Briefly, about 200 mg DM of the biomass sample were weighed and placed into 100 ml glass syringes in which buffered rumen fluid (30 ml) was pipetted. After mixing the feed with rumen fluid in the syringe, 1 ml of standard tannin solutions was injected into the syringe. The syringe was then placed in water bath at 39 °C. Incubation was carried out in duplicate within each run and each run was replicated, providing 4 replicates per treatment. Gas production was assessed until 72 h and expressed as ml/g DM.

The metabolizable energy (ME, MJ/kg DM) was calculated using the equations for forage feeds described by Menke and Steingass (1988).

### Performances and slaughtering data

In respect to the EU legislation (2009), the animals were slaughtered after fasting for 12 h, with free access to water, into a local public slaughterhouse and weighed immediately before slaughtering (slaughter weight). Slaughtering data were collected as described by Karatosidi et al. (2023); after refrigeration at 0–4 °C for further 24 h, the right-side carcass was dissected into meat cuts that were individually weighed. The *Longissimus lumborum* (Ll) muscle was removed from each right-side carcass, packed into an ethylene bag and transported into refrigerated conditions to the university laboratory.

### Physical parameters chemical composition and fatty acid analyses of meat from the Longissimus lumborum muscle

All the *Longissimus lumborum* muscles were divided into three parts: the first one was analysed upon arrival to the laboratory, i.e. 72 h post slaughtering (Day 3), while the other two were sealed in plastic bags, vacuum-packed and aged for 6 (Day 9) or 11 days (Day 14) at 0–4 °C. At all the three ageing times, meat samples were assessed for physical, chemical, and fatty acid profile and malondialdehyde (MDA) concentration.

The Ll samples were assessed for pH, colour indices and meat tenderness as reported by Tarricone et al. (2019). Homogeneous pieces of meat (approximately 5 cm thick) were cut from the Ll sample and weighed before and after cooking in a ventilated electric oven until an internal temperature of 75 °C to calculate the percentage of water loss (ASPA, 1996).

*Longissimus lumborum* muscle samples, stored at 4 °C for 3, 9 and 14 days, were analysed to evaluate the lipid oxidation through the measuring of 2-thiobarbituric acid reactive substances concentration (T-BARS) and expressed as mg MDA/kg meat (Salih et al. 1987).

Chemical analysis and fatty acids (FAs) profile were performed as previously described in Karatosidi et al. (2023) on all raw samples of the Ll muscle. The conjugated linoleic acid (CLA) content in meat was assessed as previously described by Caputi Jambrenghi et al. (2009). The atherogenic (AI) and thrombogenic (TI) indices from the fatty acid composition were calculated according to Ulbricht and Southgate (1991).

### Statistical analysis

Data were analysed using the SAS^®^ software (SAS Institute Inc., Cary, North Carolina, USA) (2002). Data referring to chemical composition, fatty acid profile and *in vitro* gas production of biomass were subjected to analysis of variance (ANOVA) with type of grazing (grassland vs wood-pasture), month of sampling and their interactions as factors.

Data on animal performance and slaughtering data were subjected to ANOVA with type of pasture (*P*), age (*A*) at slaughter (14 vs 18 months) and their interactions as factors.

Meat quality traits were analysed by ANOVA for repeated measures with type of pasture (*P*) and age (*A*) at slaughter as non-repeated factors, while ageing time (*T*) and their interaction as repeated factors. In Tables 8–10, since the interactions *P* × *A*, *A* × *T* and *P* × *T* were not significant, the interaction *P* × *A* × *T* was not reported.

**Table 8.** Physical characteristics of the *Longissimus lumborum* muscle in Podolian young bulls in relation to the pasture ­system, the age at slaughter and meat ageing time.

Pasture system	Wood pasture						Grass						SEM	Effects					
Age at slaughter (months)	14			18			14			18									
Ageing time (days)	3	9	14	3	9	14	3	9	14	3	9	14		*P*	*A*	*T*	*P × A*	*A × T*	*P × T*
pH	5.31	5.36	5.33	5.45	5.42	5.29	5.77	5.76	5.39	5.63	5.53	5.42	0.138	0.124	0.074	0.266	0.199	0.309	0.246
L*	36.04	38.58	42.04	37.16	38.54	39.91	35.59	36.06	41.43	35.10	36.30	38.52	0.241	0.227	0.151	0.062	0.174	0.090	0.341
a*	13.33	14.36	15.52	13.35	14.25	16.69	13.74	15.45	16.48	13.06	14.87	16.08	2.815	0.206	0.637	0.013	0.418	0.113	0.455
b*	13.90	13.64	14.17	12.75	11.86	11.80	11.73	12.68	12.58	11.93	11.39	13.12	1.794	0.701	0.114	0.549	0.526	0.664	0.587
WBS raw (kg/cm^2^)	1.78	1.69	1.31	2.21	2.13	2.07	2.34	2.08	1.46	2.60	2.45	1.84	1.939	0.131	0.017	0.001	0.219	0.025	0.362
WBS cooked (kg/cm^2^)	7.65	7.02	5.55	6.99	4.92	4.32	5.34	3.89	1.82	5.01	3.76	2.81	0.528	0.417	0.019	0.004	0.541	0.032	0.337
Cooking loss (%)	28.98	22.48	35.24	17.40	26.50	21.00	24.46	23.65	23.25	31.70	29.55	27.25	2.684	0.098	0.841	0.699	0.121	0.885	0.155
MDA (mg/kg of meat)	0.12	0.19	0.33	0.17	0.24	0.35	0.14	0.22	0.30	0.16	0.24	0.40	0.231	0.052	0.076	0.017	0.223	0.469	0.288

SEM standard error of means; *P* pasture system; *A* age at slaughter; *T* meat ageing time; *P* × *A*, *A* × *T*, *P* × *T* = interactions.

**Table 9.** Chemical composition (%) of the *Longissimus lumborum* muscle in Podolian young bulls in relation to the pasture system, the age at slaughter and meat ageing time.

Pasture system	Wood pasture						Grass						SEM	Effects					
Age at slaughter (months)	14			18			14			18				*P*	*A*	*T*	*P × A*	*A × T*	*P × T*
Ageing time (days)	3	9	14	3	9	14	3	9	14	3	9	14							
Moisture	75.01	75.07	74.72	74.27	74.48	74.17	74.71	72.55	73.86	74.83	73.59	74.01	1.400	0.114	0.212	0.744	0.532	0.288	0.332
Protein	21.27	21.53	21.92	21.83	21.93	22.20	21.18	22.28	22.38	21.45	21.63	22.51	0.950	0.234	0.078	0.319	0.471	0.989	0.468
Fat	1.23	1.22	0.95	2.13	1.93	1.85	1.27	1.30	1.47	1.32	1.37	1.99	0.857	0.217	0.213	0.126	0.325	0.067	0.721
Ash	1.25	1.24	1.25	1.16	1.08	1.33	1.48	1.69	1.52	1.20	1.76	0.97	0.944	0.118	0.900	0.057	0.222	0.140	0.399
N-free extracts	1.26	0.94	1.16	0.61	0.59	0.45	1.36	0.88	0.78	1.21	1.65	0.52	0.877	0.098	0.111	0.096	0.431	0.521	0.258

SEM standard error of means; *P* pasture system; *A* age at slaughter; *T* meat ageing time; *P* × *A*, *A* × *T*, *P* × *T* = interactions.

**Table 10.** Fatty acid profile (% FAME) of the *Longissimus lumborum* muscle in Podolian young bulls in relation to the pasture system, the age at slaughter and meat ageing time.

Pasture system	Wood pasture						Grass						SEM	Effects					
Age at slaughter (months)	14			18			14			18				*P*	*A*	*T*	*P × A*	*A × T*	*P × T*
Ageing time (days)	3	9	14	3	9	14	3	9	14	3	9	14							
C10:0	0.16	0.10	0.10	0.07	0.07	0.08	0.11	0.12	0.12	0.10	0.10	0.10	0.076	0.076	0.001	0.577	0.211	0.417	0.338
C12:0	0.14	0.12	0.16	0.11	0.10	0.11	0.05	0.06	0.07	0.07	0.06	0.06	0.030	0.321	0.061	0.815	0.776	0.892	0.178
C14:0	3.95	4.05	4.17	3.84	3.93	3.98	3.32	3.57	3.96	3.22	3.45	3.82	0.828	0.226	0.841	0.699	0.322	0.885	0.221
C15:0	0.53	0.55	0.57	0.48	0.45	0.44	0.47	0.45	0.45	0.34	0.32	0.30	0.101	0.463	0.001	0.812	0.767	0.891	0.534
C16:0	27.99	27.89	27.85	27.42	27.63	27.76	27.43	27.45	27.45	27.29	27.28	27.09	2.192	0.454	0.003	0.880	0.667	0.971	0.748
C17:0	0.91	0.93	0.95	0.89	0.86	0.87	1.04	1.01	1.01	0.96	0.95	0.97	0.139	0.768	0.001	0.166	0.237	0.419	0.376
C18:0	17.13	17.69	17.82	16.92	17.38	17.47	17.4	17.46	17.56	17.38	17.77	18.02	2.374	0.237	0.019	0.871	0.299	0.933	0.634
C20:0	0.08	0.09	0.09	0.08	0.09	0.13	0.12	0.08	0.11	0.13	0.11	0.13	0.023	0.105	0.064	0.588	0.264	0.767	0.299
∑ SFA	50.89	51.42	51.71	49.81	50.51	50.84	49.94	50.2	50.73	49.49	50.04	50.49	3.791	0.072	0.134	0.771	0.257	0.478	0.389
C14:1	0.49	0.47	0.43	0.62	0.57	0.54	0.49	0.47	0.41	0.48	0.44	0.38	0.219	0.137	0.138	0.654	0.178	0.824	0.734
C15:1	0.22	0.21	0.22	0.22	0.19	0.20	0.15	0.20	0.15	0.18	0.17	0.17	0.041	0.206	0.100	0.679	0.368	0.621	0.572
C16:1 n7	3.79	3.16	3.02	3.64	3.45	3.32	3.99	3.57	3.06	3.56	3.51	3.25	0.672	0.078	0.015	0.905	0.136	0.934	0.975
C17:1	0.59	0.60	0.51	0.68	0.64	0.65	0.69	0.69	0.64	0.42	0.45	0.35	0.158	0.091	0.001	0.433	0.102	0.563	0.579
C18:1 n7	1.02	1.05	1.07	1.27	1.26	1.24	1.38	1.35	1.31	1.13	1.10	1.09	0.211	0.264	0.001	0.463	0.307	0.874	0.762
C18:1 n9 *t*	0.28	0.23	0.23	0.33	0.37	0.25	0.47	0.44	0.44	0.27	0.33	0.35	0.280	0.078	0.001	0.466	0.154	0.500	0.611
C18:1 n9 *c*	36.53	36.34	36.02	37.6	37.04	37.09	36.44	36.39	36.27	36.74	36.56	36.24	3.416	0.082	0.001	0.477	0.137	0.990	0.867
C20:1 n9	0.10	0.10	0.10	0.13	0.11	0.10	0.15	0.14	0.14	0.19	0.19	0.19	0.202	0.261	0.001	0.902	0.328	0.978	0.878
∑ MUFA	43.02	42.16	41.6	44.49	43.63	43.39	43.76	43.25	42.42	42.97	42.75	42.02	3.698	0.372	0.001	0.450	0.479	0.787	0.882
C18:2 n6 *t*	0.15	0.15	0.12	0.2	0.19	0.22	0.19	0.17	0.19	0.19	0.21	0.20	0.075	0.337	0.001	0.752	0.571	0.896	0.794
C18:2 n6 *c*	2.74	2.43	2.30	2.81	2.65	2.52	2.55	2.39	2.23	2.40	2.30	2.28	0.427	0.261	0.001	0.954	0.786	0.995	0.915
CLA _c9. t11_	0.43	0.42	0.39	0.48	0.45	0.44	0.41	0.32	0.32	0.30	0.28	0.28	0.068	0.351	0.002	0.126	0.479	0.309	0.899
CLA _t10. c12_	0.07	0.07	0.06	0.10	0.09	0.09	0.11	0.10	0.10	0.10	0.10	0.10	0.022	0.634	0.001	0.151	0.724	0.162	0.682
C18:3 n3	0.21	0.21	0.21	0.26	0.27	0.26	0.51	0.55	0.51	0.43	0.4	0.42	0.066	0.225	0.001	0.942	0.467	0.995	0.733
C18:3 n6	0.06	0.07	0.06	0.09	0.09	0.09	0.10	0.09	0.09	0.09	0.09	0.08	0.023	0.472	0.001	0.775	0.588	0.954	0.917
C20:2 n6	0.05	0.04	0.02	0.04	0.03	0.01	0.03	0.20	0.01	0.04	0.02	0.02	0.018	0.328	0.007	0.260	0.481	0.017	0.239
C20:3 n3	0.30	0.26	0.25	0.35	0.30	0.24	0.36	0.29	0.18	0.29	0.27	0.23	0.112	0.374	0.001	0.596	0.626	0.524	0.734
C20:3 n6	0.08	0.08	0.07	0.11	0.12	0.12	0.11	0.10	0.10	0.10	0.06	0.04	0.037	0.088	0.001	0.541	0.624	0.614	0.831
C20:5 n3	0.03	0.02	0.01	0.02	0.01	0.03	0.05	0.04	0.08	0.05	0.04	0.04	0.091	0.429	0.001	0.079	0.372	0.181	0.732
C21:5 n3	0.00	0.00	0.01	0.03	0.06	0.05	0.09	0.10	0.06	0.01	0.00	0.02	0.024	0.121	0.001	0.280	0.269	0.464	0.399
C22:5 n3	0.04	0.04	0.05	0.05	0.09	0.07	0.07	0.09	0.11	0.09	0.09	0.08	0.035	0.063	0.001	0.370	0.639	0.365	0.734
C22:6 n6	0.02	0.03	0.04	0.02	0.01	0.04	0.00	0.02	0.02	0.03	0.02	0.02	0.018	0.327	0.983	0.375	0.362	0.419	0.645
∑ PUFA	4.18	3.82	3.59	4.56	4.36	4.18	4.58	4.46	4.00	4.12	3.88	3.81	0.646	0.267	0.001	0.820	0.621	0.765	0.389
∑ UFA	47.2	45.98	45.19	49.05	47.99	47.57	48.34	47.71	46.42	47.09	46.63	45.83	3.897	0.471	0.134	0.771	0.449	0.478	0.873
Other	1.91	2.60	3.10	1.14	1.50	1.59	1.72	2.09	2.85	3.42	3.33	3.68	0.074	0.376	0.100	0.679	0.478	0.621	0.682
n-6	3.60	3.29	3.06	3.85	3.63	3.53	3.50	3.39	3.06	3.25	3.08	3.02	0.455	0.274	0.001	0.992	0.391	0.994	0.737
n-3	0.58	0.53	0.53	0.71	0.73	0.65	1.08	1.07	0.94	0.87	0.8	0.79	0.181	0.329	0.001	0.586	0.582	0.640	0.899
n-6/n-3	6.21	6.21	5.77	5.42	4.97	5.43	3.24	3.17	3.26	3.74	3.85	3.82	1.019	0.652	0.001	0.776	0.619	0.885	0.792
A.I.	0.91	0.89	0.87	0.85	0.75	0.8	0.71	0.77	0.79	0.74	0.68	0.68	0.173	0.361	0.705	0.733	0.698	0.498	0.691
T.I.	1.44	1.44	1.43	1.29	1.21	1.25	1.29	1.32	1.38	1.39	1.35	1.41	0.314	0.312	0.001	0.670	0.635	0.569	0.819
UFA/SFA	0.93	0.89	0.87	0.98	0.95	0.94	0.97	0.95	0.92	0.95	0.93	0.91	0.157	0.268	0.138	0.654	0.923	0.824	0.991
SFA/PUFA	0.08	0.07	0.07	0.09	0.09	0.08	0.09	0.09	0.08	0.08	0.08	0.08	3.277	0.244	0.914	0.275	0.728	0.529	0.943

SEM standard error of means; *P* pasture system; *A* age at slaughter; *T* meat ageing time; *P* × *A*, *A* × *T*, *P* × *T* interactions; SFA saturated fatty acids; MUFA mono unsaturated fatty acids; PUFA poly unsaturated fatty acids; UFA unsaturated fatty acids; A.I. atherogenic index; T.I. thrombogenic index.

Results are reported as means and standard errors of the means (SEM). When significant effects were found at *p* < 0.05, means were compared using Student’s *t* test.

## Results

### Chemical composition, fatty acid profile and metabolizable energy of the two pastures

Table 4 shows the chemical composition and the fatty acid profile of the two pastures. Crude fibre and acid detergent fibre (ADF) values were higher (*p* < 0.05) in grass samples collected in April, June, August, and October. The month of sampling significantly affected the chemical composition of pasture: in February, both types of pasture had less (*p* < 0.05) DM, crude fibre and NDF, while protein and ash contents were markedly higher (*p* < 0.05).

**Table 4.** Chemical composition and fatty acid profile of wood pastures and grasses collected during the year.

Pasture system	Wood pasture					Grass					SEM	Effects		
Month	February	April	June	August	October	February	April	June	August	October		*P*	*M*	*P ×* *M*
	**Chemical composition**													
Dry matter (%)	32.00	36.40	53.20	63.80	41.80	32.40	34.60	59.40	69.40	42.60	1.58	0.412	0.025	0.267
Protein (% on DM)	12.84	11.28	9.30	7.40	10.02	15.60	13.00	9.99	7.11	10.49	1.04	0.112	0.017	0.074
Ether extract (% on DM)	2.01	2.77	2.89	0.93	1.25	2.28	2.44	2.88	1.04	1.84	0.32	0.437	0.147	0.261
Ash (% on DM)	17.77	13.17	10.08	8.25	17.59	19.08	14.18	11.22	13.61	21.02	1.05	0.281	0.040	0.374
Crude fibre (% on DM)	20.55	21.65	34.55	39.52	30.28	20.31	25.82	38.41	40.05	27.23	2.10	0.041	0.026	0.037
NDF (% on DM)	52.75	55.49	61.33	69.45	55.33	55.06	55.04	62.92	70.39	52.14	1.17	0.223	0.034	0.088
ADF (% on DM)	27.00	27.92	40.54	48.63	46.95	28.11	34.44	43.66	47.80	49.17	1.14	0.047	0.041	0.095
**Fatty acid profile (% fatty acid methyl esters)**														
n-6	15.16	15.59	15.14	10.71	14.25	19.56	22.52	19.28	12.59	21.93	0.28	0.002	0.007	0.025
n-3	20.20	20.38	20.45	11.29	18.70	22.16	26.08	20.69	10.96	16.06	1.45	0.004	0.006	0.044
MUFA	37.64	34.75	34.55	22.66	32.18	31.57	29.70	30.03	28.08	30.10	1.11	0.004	0.007	0.045
PUFA	35.36	35.97	35.59	22.00	32.95	41.72	48.00	39.97	23.55	37.99	0.87	0.005	0.003	0.041
SFA	27.00	29.28	29.86	55.34	34.87	26.71	22.30	28.00	48.37	31.91	0.52	0.224	0.008	0.312

SEM standard error of means; *P* pasture system; *M* month of sampling; *P* × *M* interaction; NDF neutral detergent fibre; ADF acid detergent fibre; MUFA mono unsaturated fatty acids; PUFA poly unsaturated fatty acids; SFA saturated fatty acids.

Significant differences between the two pasture systems were observed for the fatty acid profile (*p* < 0.01). n-6 fatty acids and Poly Unsaturated Fatty Acids (PUFAs) were higher (*p* < 0.05) in grass samples collected during all the months, while n-3 fatty acids were higher in grass samples collected during February and April. Mono Unsaturated Fatty Acids (MUFAs) were higher in the wood pasture samples collected in February, April, June and October. Samples collected in August showed lower (*p* < 0.01) values of n-6, n-3, MUFAs and total PUFAs.

Differences in the chemical composition of pasture affected the nutritive value of biomass (Table 5); in fact, a higher production of gas was found in April and June for both pasture systems, with significant differences for the effects of pasture, month of sampling and their interaction (*p* < 0.01). The same trend was observed for metabolizable energy, that was higher (*p* < 0.05) for both types of pasture collected during April and June.

**Table 5.** Kinetics of gas production and nutritive value of wood pastures and grasses collected during the year.

Pasture system	Wood Pasture					Grass						Effects		
Month	February	April	June	August	October	February	April	June	August	October		SEM	*P*	*M*	*P* × *M*
GP^3^ (ml/200 mg DM)	30.41	41.14	35.60	31.63	36.01	39.05	44.67	44.84	37.39	39.23	9.47	0.002	0.006	0.012
ME^4^ (MJ/kg DM)	6.72	8.51	7.10	6.18	6.20	7.96	8.55	8.44	7.24	6.16	0.26	0.017	0.042	0.089

SEM standard error of means; *P* pasture system; *M* month of sampling; *P* × *M* interaction; GP gas production; ME metabolizable energy.

### Slaughtering and carcass traits of young bulls

The results concerning growth performances and slaughtering data of Podolian young bulls are shown in Table 6. The slaughter weight was significantly affected by the pasture system (*p* < 0.05) and by the age at slaughter (*p* < 0.01), along with the interaction of the two effects (*P* × *A*: *p* < 0.05). Young bulls raised on grass showed significantly greater slaughter weights as compared to those fed on woodland, at both the slaughtering ages (*p* < 0.05).

**Table 6.** Slaughtering data of Podolian young bulls in relation to the pasture system and the age at slaughter.

Pasture system	Wood Pasture		Grass		SEM	Effects		
Age at slaughter (months)	14	18	14	18		*P*	*A*	*P* × *A*
Initial weight (kg)	342.70	339.46	341.85	343.33	7.592	0.125	0.247	0.124
Slaughter weight (kg)	411.10	501.00	433.33	537.75	33.969	0.039	0.001	0.022
ADG^3^ (kg/d)	0.57	0.67	0.76	0.81	0.207	0.044	0.058	0.112
Hot carcass weight (kg)	213.95	269.18	235.49	315.26	6.952	0.018	0.001	0.021
Cold carcass weight (kg	211.40	265.18	230.53	307.00	7.042	0.024	0.001	0.029
Drip loss (%)	1.19	1.53	2.11	2.62	0.533	0.049	0.053	0.097
Hot dressing percentage (%)	52.04	53.73	54.41	58.62	3.206	0.067	0.057	0.314
Cold dressing percentage (%)	51.42	52.93	53.20	57.09	2.350	0.051	0.053	0.451

SEM standard error of means; *P* pasture system; *A* age at slaughter; *P* × *A* interaction; ADG average daily gain.

The average daily gain (ADG) of young bulls was not affected by the slaughtering age, while it was greater following grass feeding (*p* < 0.05).

The hot and cold carcass weights were lower (*p* < 0.01) when young bulls were slaughtered at 14 months, regardless of the pasture system. Young bulls raised on grassland showed a greater hot (*p* < 0.01) and cold carcass (*p* < 0.05) weight. The drip loss following carcass refrigeration for 24 h was lower (*p* < 0.05) in young bulls grazing on woodland. The hot and cold dressing percentages were not affected neither by the pasture system, nor by the age at slaughter of young bulls.

The dissection data of the right-side carcass of Podolian young bulls are presented in Table 7. The right-side carcass weight was significantly greater in 18 months old bulls as compared to 14 months (*p* < 0.01). Young bulls grazing on grassland showed heavier right-side carcasses in comparison with those raised on woodland (*p* < 0.01). Slaughtering at 14 months determined a significantly higher incidence of the round, the chuck and the shoulder clod, while at 18 months a greater proportion (*p* < 0.01) of the loin, the briskets, and the flank was recorded.

**Table 7.** Dissection data (%) of the right-side carcass of Podolian young bulls in relation to the pasture system and the age at slaughter.

Pasture system	Wood Pasture		Grass		SEM	Effects		
Age at slaughter (months)	14	18	14	18		*P*	*A*	*P* × *A*
Right-side weight (kg)	105.65	137.83	115.98	156.25	6.745	0.031	0.001	0.021
Round	30.41	27.40	31.72	26.97	1.600	0.074	0.001	0.934
Rib + Loin	8.72	9.99	6.66	9.11	1.162	0.071	0.002	0.563
Tender loin	1.96	1.91	1.90	1.91	0.426	0.174	0.088	0.874
Brisket	14.15	17.04	15.79	17.55	2.000	0.067	0.001	0.500
Shoulder clod	16.50	15.17	15.22	14.67	0.942	0.144	0.001	0.990
Chuck	15.33	14.14	15.39	13.76	1.769	0.077	0.012	0.978
Flank	10.80	12.23	11.12	13.25	1.523	0.068	0.001	0.787

SEM standard error of means; *P* pasture system; *A* age at slaughter; *P* × *A* = interaction.

### Physical properties of meat from the Longissimus lumborum muscle

The physical characteristics of the Ll muscle of Podolian young bulls are reported in Table 8. No significant differences were observed for meat pH, L*, a* and b* values, accordingly to other findings (Sacarrão-Birrento et al. 2022). In our study, neither the pasture system nor the age at slaughter affected the pH values; the only difference in meat colour was observed following ageing, that led to a significant increase of the red index (*p* < 0.05) during storage.

The Warner–Bratzler Shear (WBS) force values for raw and cooked meat were unaffected by the pasture system but the shear force decreased (*p* < 0.01) in relation to the ageing time in all the groups. Furthermore, meat tenderness was greater in young bulls slaughtered at 14 months (*p* < 0.01) as compared to the older counterparts.

No significant differences among groups were observed for meat cooking loss. The ageing time markedly (*p* < 0.05) increased the MDA concentration from 3 to 14 days of storage, regardless of the pasture system and the slaughtering age.

### Chemical composition and fatty acid profile of meat from the Longissimus lumborum muscle

The chemical composition of meat from the Ll muscle was unaffected by the pasture system, age of slaughter and ageing time, as shown in Table 9.

Table 10 shows the fatty acid profile of the Ll muscle in Podolian cattle in relation to the pasture system, age at slaughter and ageing time. No significant differences were found among groups for any of the effects studied. The concentration of total SFA slightly increased during ageing, probably due to the saturation of MUFAs and PUFAs that decreased during storage.

Finally, the n-6/n-3 ratio, and the thrombogenic and atherogenic indices were not influenced by the effects taken into consideration in this study.

## Discussion

Pasture quality progressively decreased during the sampling period. The low crude protein and the high crude fibre contents found during the June–August period is in line with the botanic composition of the grass during summer (Ritz et al. 2020). These results confirm that herbage maturity is the most critical factor influencing forage quality (Buxton 2012). In general, in late spring, the forage quality declines due to the increased environmental temperature that leads to advanced plant maturity (Van Soest 2018). As expected for a typical Mediterranean wood pasture, we recorded a gradual increase of the fibre content from February to August, followed by a drop in October. In agreement with our results, Ripamonti et al. (2023) found significant changes in pasture production and nutritive value during the year in Tuscany. The chemical composition of feeds does not provide sufficient information on their real nutritive value. The animals’ ability to utilize feed nutrients has a significant impact on their growth performances. The *in vitro* gas production system helps to better quantify nutrient utilization (Getachew et al. 2004).

As for the chemical composition of pastures, biomass collected in February and April showed better nutritive value due to their high-protein and low-fibre contents, which positively affected the productivity of ruminant livestock (Lima et al. 2018). It has been reported that rumen microbiota requires a minimum crude protein of 70–80 (g/kg DM) to optimize the breakdown of cell wall components, otherwise, in a ruminant production system based on grassland with crude protein content lower than 70 g/kg DM, as found in our samples collected during June and August, nitrogen supplementation may be taken into consideration in order to improve the feed intake and digestibility (Sampaio et al. 2010). The moderate dietary energy concentration found in this trial, although below 10.5 MJ/kg DM, that is low for high potential beef genotypes (Hessle et al. 2019), may be considered suitable for growth in Podolian cattle, that is an autochthonous breed well adapted to natural pastures low in nutritive value.

The ADG values recorded in our study are lower than those reported by other authors for Podolian young bulls slaughtered at 15 (Marino et al. 2006b) and 18 months of age (Braghieri et al. 2011). Pogorzelska-Przybyłek et al. (2018) did not find any differences in the average daily gain and dressing percentage in crossbred bulls and steers slaughtered at 18 or 21 months; in this study, the dressing percentage found for Podolian young bulls raised on grassland and slaughtered at 18 months (58.62%) was similar to their results (57.70%).

The type of pasture significantly affected only the weight of the right-side carcass, while no effect was observed on the proportion of single meat cuts. The results obtained in this study are slightly lower as compared to those reported by other authors (Marino et al. 2006b, Braghieri et al. 2004; Vicenti et al. 2009) in young bulls of the same breed that underwent a finishing period with high levels of concentrate. Young bulls fed on grassland showed heavier right-side carcasses in comparison with those raised on woodland, and the interaction between the pasture and age effect was also significant. This result may be attributable to the higher metabolizable energy of the grassland as compared to the silvo-pastoral biomass. Differences in the carcass weight and conformation, and in the proportion of valuable meat cuts in relation to the age of slaughter has been reported also by other authors (Dayton and White 2008; Duckett et al. 2014; Pogorzelska-Przybyłek et al. 2018). These differences may be attributable to changes in steroid concentration, mainly testosterone, that occurs during growth; testosterone binds to muscle receptors and stimulates amino acid incorporation into proteins, leading to an increase in muscle weight but not in fat deposition (Dayton and White 2008), as may have occurred in Podolian young bulls characterized by lean muscular mass.

In the present study, the pH values were all below 5.8, which is the recommended value for beef subjected to ageing (Sakowski et al. 2022). The effects of feeding and slaughtering age on beef meat colour have been thoroughly investigated, although with controversial results. In beef, meat colour is one of the most important quality attributes assessed visually by consumers along with marbling. It has been reported that older animals show higher myoglobin and iron levels, that lead to a darker meat colour, and that meat from animals raised on grassland has a bright red colour as compared to feedlot counterparts (French et al. 2000, 2001). Bureš and Bartoň (2012) documented that extending the slaughter age in cattle from 14 to 18 months, as we did in this study, did not affect the L* value but determined a higher redness index in meat from older animals. Several authors stated that meat colour, tenderness, and shelf life are affected by pH (Ahnström et al. 2009; Filipčík et al. 2009); in our study, neither the pasture system nor the age at slaughter affected the pH values of meat; the only difference in meat colour was observed following ageing, where the red index markedly increased over time, as also found by other authors (Pogorzelska-Przybyłek et al. 2018). Ultimate meat tenderness is highly variable and an important factor in predicting the eating quality of beef. Previous studies documented that tenderness may be affected by animal intrinsic factors such as genotype, sex, and slaughtering age as well as by extrinsic ones, mainly the feeding system (French et al. 2000, Ivanov 2023). Differences in shear force between raw and cooked samples has been reported and related to the muscle structure, water holding ability and collagen content (Foraker et al. 2020). There is general agreement on the effect of ageing on beef meat tenderization; De Huidobro et al. (2003) reported that shear force tended to decrease in raw but not in cooked meat. Conversely, in our study meat tenderness was positively and significantly affected by ageing time, more in cooked than in raw meat samples. Furthermore, in this study, the WBS values for cooked meat samples were greater than raw ones, as compared to previous trials carried out on Podolian young bulls (Ragni et al. 2014; Tarricone et al. 2019). Meat tenderization through ageing involves several features that lead to complex changes in muscle metabolism, among which myofibrillar fragmentation and enzymatic proteolysis of muscular fibres (Christensen et al. 2004). Cattle beef with shearing force below 5 kg/cm^2^ is perceived as being tender (De Menezes et al. 2019); therefore, WBS values of meat samples collected from grassland animals at both slaughtering ages fell within the range of beef acceptability. The reaction of MDA with TBA has been extensively used to detect the oxidative deterioration of lipids in muscle foods (Marino et al. 2014). In our study, we observed a significant increase of MDA concentration especially from 9 to 14 days of ageing, in accordance with another study carried out in the same breed (Braghieri et al. 2004). Our results show that the concentration of MDA found in meat samples from all groups, ranged from 0.12 to 0.40 that is far below the value of 1–2 mg/kg of meat considered as the threshold value for rancidity (Reitznerová et al. 2017).

The chemical composition of meat from the Ll muscle was unaffected by the pasture system, age of slaughter and ageing time. Other authors have reported that intramuscular fat concentration increases as carcass weight increased (Bureš and Bartoň 2012; Pogorzelska-Przybyłek et al. 2018). Many factors affect intramuscular fat content among which breed, feeding, age and sex (Ponnampalam et al. 2024). Podolian cattle have naturally lean meat, especially at the slaughter ages tested in our study. Furthermore, the lack of significance observed for meat chemical composition may also be due to statistical sensitivity.

In this study, the proportion of SFA, MUFA and PUFA observed in all the groups were similar to those reported by Vicenti et al. (2009). Braghieri et al. (2004) found a higher concentration of PUFA and a lower amount of MUFA as compared to our results; it may be hypothesised that a diet rich in grass and seeds, present in wood pasture, may have increased the MUFA content in meat samples. The n-6/n-3 ratio was markedly lower in meat from grassland animals, regardless of the age of slaughter. The recommended value of this ratio for human nutrition should not exceed 10:1, while in our study the n-6/n-3 ratio fell within the range between 3.17 and 6.21 (Ragni et al. 2014). The PUFA/SFA ratio, considered to be an important marker for the nutritional value of foods for the human diet, should be about 0.45–0.65 (Department of Health and Social Security 1984), while a lower ratio in the diet may increase the incidence of cardiovascular disease. The PUFA/SFA ratio in ruminant meat has been reported to be low due to the biohydrogenation of the unsaturated fatty acids assumed by the diet; in this study, this ratio was low but comparable to the values reported by other authors (Vicenti et al. 2009).

In conclusion, Podolian young bulls raised on grassland and slaughtered at 18 months showed better growth performances and yield of meat cuts, but no differences were found between pasture systems as for meat chemical and physical quality. Furthermore, ageing for 9 days positively affected meat WBS without increasing MDA concentration.

## Data Availability

All data generated or analyzed during this study are included in this article.
